# Contribution of NAADP to Glutamate-Evoked Changes in Ca^2+^ Homeostasis in Mouse Hippocampal Neurons

**DOI:** 10.3389/fcell.2020.00496

**Published:** 2020-06-25

**Authors:** Julia Hermann, Melanie Bender, Dagmar Schumacher, Marcel S. Woo, Artem Shaposhnykov, Sina C. Rosenkranz, Vladimir Kuryshev, Chris Meier, Andreas H. Guse, Manuel A. Friese, Marc Freichel, Volodymyr Tsvilovskyy

**Affiliations:** ^1^Institute of Pharmacology, Heidelberg University, Heidelberg, Germany; ^2^DZHK (German Centre for Cardiovascular Research), Partner Site Heidelberg/Mannheim, Heidelberg, Germany; ^3^Institut für Neuroimmunologie und Multiple Sklerose, Universitätsklinikum Hamburg-Eppendorf, Hamburg, Germany; ^4^Organic Chemistry, Department of Chemistry, University of Hamburg, Hamburg, Germany; ^5^The Calcium Signaling Group, Department of Biochemistry and Molecular Cell Biology, University Medical Center Hamburg-Eppendorf, Hamburg, Germany

**Keywords:** hippocampal neurons, glutamate, NAADP, Ca^2+^ homeostasis, neuronal excitotoxicity

## Abstract

Nicotinic acid adenine dinucleotide phosphate (NAADP) is a second messenger that evokes calcium release from intracellular organelles by the engagement of calcium release channels, including members of the Transient Receptor Potential (TRP) family, such as TRPML1, the (structurally) related Two Pore Channel type 1 (TPC1) and TPC2 channels as well as Ryanodine Receptors type 1 (RYR1; Guse, 2012). NAADP evokes calcium release from acidic calcium stores of many cell types (Guse, 2012), and NAADP-sensitive Ca^2+^ stores have been described in hippocampal neurons of the rat (Bak et al., 1999; McGuinness et al., 2007). Glutamate triggers Ca^2+^-mediated neuronal excitotoxicity in inflammation-induced neurodegenerative pathologies such as Multiple Sclerosis (MS; Friese et al., 2014), and when applied extracellularly to neurons glutamate can elevate NAADP levels in these cells. Accordingly, glutamate-evoked Ca^2+^ signals from intracellular organelles were inhibited by preventing organelle acidification (Pandey et al., 2009). Analysis of reported RNA sequencing experiments of cultured hippocampal neurons revealed the abundance of Mcoln1 (encoding TRPML1), Tpcn1, and Tpcn2 (encoding TPC1 and TPC2, respectively) as potential NAADP target channels in these cells. Transcripts encoding Ryr1 were not found in contrast to Ryr2 and Ryr3. To study the contribution of NAADP signaling to glutamate-evoked calcium transients in murine hippocampal neurons we used the NAADP antagonists Ned-19 (Naylor et al., 2009) and BZ194 (Dammermann et al., 2009). Our results show that both NAADP antagonists significantly reduce glutamate-evoked calcium transients. In addition to extracellular glutamate application, we studied synchronized calcium oscillations in the cells of the neuronal cultures evoked by addition of the GABA_A_ receptor antagonist bicuculline. Pretreatment with Ned-19 (50 μM) or BZ194 (100 μM) led to an increase in the frequency of bicuculline-induced calcium oscillations at the cost of calcium transient amplitudes. Interestingly, Ned-19 triggered a rise in intracellular calcium concentrations 25 min after bicuculline stimulation, leading to the question whether NAADP acts as a neuroprotective messenger in hippocampal neurons. Taken together, our results are in agreement with the concept that NAADP signaling significantly contributes to glutamate evoked Ca^2+^ rise in hippocampal neurons and to the amplitude and frequency of synchronized Ca^2+^ oscillations triggered by spontaneous glutamate release events.

## Introduction

In neurons, changes of free intracellular calcium ion concentration [(Ca^2+^)_i_] regulate many physiological functions such as neuronal plasticity, gene transcription and synaptic transmission (Ureshino et al., 2019). Defects in Ca^2+^ homeostasis are a reasonable cause for cell death and, eventually, neurodegeneration (Wojda et al., 2008; Szydlowska and Tymianski, 2010). Under these conditions, the homeostatic system, which assures low [Ca^2+^]_i_ in the range of 100 nM (in resting conditions), is defective (Nedergaard and Verkhratsky, 2010; Zundorf and Reiser, 2011). The tight equilibrium in [Ca^2+^]_i_ is usually maintained by a large number of ion channels and transporters in the plasma membrane as well as in the intracellular compartments of the cell.

Exaggerated rise in [Ca^2+^]_i_, which can be triggered by glutamate accumulation, e.g., after hypoxia, leads to apoptosis and necrotic cell death. Glutamate-evoked changes in intracellular Ca^2+^ homeostasis involve the activation of ionotropic NMDA, AMPA, and kainate (KA) receptors, as well as the mechanisms downstream of the activation of metabotropic glutamate receptors (mGluRs; Reiner and Levitz, 2018). The underlying processes of Ca^2+^-overload mediated toxicity may include over-activation of several types of enzymes, e.g., proteases of the calpain family, phosphatase calcineurin, nitric oxide synthases, endonucleases (leading to DNA fragmentation), and phospholipid scramblase leading to phosphatidyl-choline PS exposure in the outer leaflet of the plasma membrane (Orrenius et al., 2003; Hardingham and Bading, 2010). In acute (e.g., following cerebral ischemia) or chronic neurodegenerative diseases, such as Alzheimer’s disease (AD), Parkinson’s disease (PD), and Multiple sclerosis (MS), glutamate elevations evoke an impairment of mitochondrial function, survival-promoting gene expression and the maintenance of structural integrity of neurons that all act in concert during the development of neurodegeneration (Bading, 2017). Impaired Ca^2+^ homeostasis in familial forms of AD is thought to occur from a dysfunction of presenilins, which act as Ca^2+^ release channel in the ER, a subsequent Ca^2+^ accumulation in the ER, and downregulation of neuronal store-operated Ca^2+^ entry (Popugaeva et al., 2017). Furthermore, Aß42/40 proteins fragments, which arise by proteolysis from the amyloid precursor protein (APP) and aggregate to neurotoxic oligomers, can form Ca^2+^-permeable pores in the plasma membrane and modulate activity of voltage dependent calcium channels and NMDA receptors. Elevated ER Ca^2+^ content leads to enhanced Ca^2+^ release via D-*myo*-inositol 1, 4, 5-trisphosphate receptors (IP_3_R), and ryanodine receptors (RYR) as well as Ca^2+^ accumulation in the cytosol (Popugaeva et al., 2017). In PD the impaired Ca^2+^ homeostasis leading to increased vulnerability in dopaminergic neurons of the substantia nigra is characterized by an activity-related oscillatory intracellular Ca^2+^ load potentially caused by the altered activity of multiple Ca^2+^ conducting channels including store-operated channels, ionotropic glutamate receptors, and voltage gated Ca_V_1 channels (Duda et al., 2016). Notably, the application of dihydropyridines as negative allosteric modulators of Ca_V_1 Ca^2+^ channels is associated with a decreased risk and progression of PD (Surmeier et al., 2017).

Glutamate-evoked neurotoxicity is also a key process in the pathogenesis of MS, and glutamate levels are elevated in the CSF and brains of MS patients. A growing body of evidence has suggested that the inflammatory insults in MS determine neurodegeneration by alterations of ion exchange mechanisms and here in particular Ca^2+^ handling (Friese et al., 2014). Dysregulation of cellular Ca^2+^ homeostasis evoked by glutamate was previously considered to be a result of Ca^2+^ entry via ionotropic glutamate receptors; however, our recent studies have identified that the neuronally expressed Na^+^- and Ca^2+^-permeable acid sensing ion channel-1 (ASIC1) and Ca^2+^-activated transient receptor potential melastatin 4 (TRPM4) crucially contribute to maladaptive cation handling under inflammatory conditions (Friese et al., 2007; Schattling et al., 2012). Specifically, our studies showed that neuronal Ca^2+^ overload with excessive activation of Ca^2+^-dependent processes may be one component of neuronal injury. However, the constituents of channels in both the plasmalemmal and intracellular organelles contributing to cytosolic Ca^2+^ rise, as well as the mechanisms by which intracellular Ca^2+^ homeostasis is orchestrated, e.g., by providing a sufficient buffering capacity during neuroinflammation, remain to be poorly understood.

The cellular Ca^2+^ homeostasis can be modulated not only by Ca^2+^ channels in the plasma membrane, but also by Ca^2+^ release from intracellular Ca^2+^ stores. Organelles that function as Ca^2+^ stores with a major impact on cytosolic Ca^2+^ homeostasis include (i) the mitochondrion, (ii) the endoplasmic reticulum, and (iii) the lysosomes that can buffer and release Ca^2+^. Over the last years, numerous studies have revealed that glutamate stimulation in neurons evokes, in addition to IP_3,_ the synthesis of Nicotinic acid adenine dinucleotide phosphate (NAADP) and Ca^2+^ release from acidic stores. NAADP-sensitive Ca^2+^ stores have been described in brain microsomes (Bak et al., 1999) and in lysosome-related organelles in hippocampal neurons of the rat (Bak et al., 1999; McGuinness et al., 2007). Extracellular glutamate application is able to elevate cellular NAADP levels in hippocampal neurons, and glutamate-evoked Ca^2+^ signals from intracellular organelles were inhibited by antagonizing acidification of lysosomes (Pandey et al., 2009). A recent study has also shown that Ca^2+^ entry via voltage gated Ca^2+^ channels triggers Ca^2+^ release from the lysosome via an NAADP-sensitive channel and, subsequently, fusion of the lysosome with the plasma membrane (Padamsey et al., 2017). In addition, metabotropic glutamate receptor 1 (mGluR1) has been reported to couple to NAADP signaling eliciting Ca^2+^ release from acidic stores via TPC channels during synaptic plasticity (Foster et al., 2018). In non-neuronal cells the NAADP-evoked Ca^2+^ release depends on ryanodine receptors, such as on Ryanodine Receptors type 1 (RYR1) in *T* cells (Diercks et al., 2018), and on TRPML1 channels in fibroblasts (Zhang et al., 2011).

In this study we aimed to investigate the contribution of NAADP signaling to glutamate evoked calcium transients in murine hippocampal neurons following extracellular glutamate application using NAADP antagonists. A second set of experiments was performed under conditions of facilitated spontaneous glutamate release events by pharmacological inhibition of GABA_A_ receptors that, while being active, exhibit an inhibitory influence on neurotransmitter exocytosis. The results of our experiments reveal that the NAADP antagonists Ned-19 (Naylor et al., 2009) and BZ194 (Dammermann et al., 2009), respectively, significantly reduced glutamate evoked calcium transients in neurons upon extracellularly applied glutamate and that these NAADP antagonists increase the frequencies of bicuculline-evoked calcium oscillations at the cost of lower calcium transient amplitudes supporting the concept that NAADP signaling significantly contributes to glutamate evoked Ca^2+^ rise in hippocampal neurons.

## Materials and Methods

### Isolation of Hippocampal Neurons From Mouse Embryos

Pregnant females (E16.5) from timed mating were euthanized by CO_2_ inhalation. Embryos were isolated after ovarian section. After removal of the placenta and the uterus the embryos were placed in a Petri dish with 25 ml ice cold Hanks’ Balanced Salt Solution (ThermoFisher). Hippocampi were dissected from the embryo’s head under a stereo microscope (8× magnification, Stemi SV4, Carl Zeiss) and digested with trypsin/EDTA (Sigma-Aldrich) and warmed to 37°C. For each independent cell preparation hippocampi from at least 4 Embryos were pooled. Cells were dissociated by repeated trituration steps using a Pasteur pipette and filtered using a cell strainer (40 μm, Corning). Thereafter, cells were centrifuged (1.700*g*, 2 min), and resuspended in Neurobasal medium (Neurobasal medium, ThermoFisher: 21103049, supplemented with 1% Penicillin- Streptomycin, 45 μM ß-Mercaptoethanol, 1× B27 Supplement, and 0.5 mM GlutaMAX-I). Cells were then plated on Poly-D-lysine coated coverslips (382.000 cells per coverslip, diameter 18 mm) and cultured in 12 well plates at 37°C and 5% CO_2_ in Neurobasal medium supplemented with 1% Penicillin- Streptomycin, 45 μM ß-Mercaptoethanol, 1 × B27 Supplement, and 0.5 mM GlutaMAX-I. At day 3 after plating, cells were treated with Ara-C (500 nM) to prevent proliferation of glial cells. Medium was changed at day 6 or 7 and microfluorimetric calcium imaging experiments were performed at day 13 to 15 *in vitro* (d.i.v.).

All animal procedures were approved and performed according to the regulations of the Regierungspräsidium Karlsruhe and the University Heidelberg (*T* 25/16) and conformed to the guidelines from the directive 2010/63/EU of the European Parliament on the protection of animals used for scientific purposes.

### Fluorimetric [Ca^2+^]_i_ Measurements

Cultured hippocampal neuronal cells were loaded with a fluorescent Ca^2+^ indicator Fura-2 by incubation in Physiological Salt Solution (PSS) supplemented with 2 μM Fura-2 AM (Thermo Fisher Scientific, Darmstadt, Germany) and 0.1% Pluronic F-127 (Sigma-Aldrich Chemie GmbH, Steinheim, Germany) for 30 min at room temperature and prior to imaging were washed with PSS. The PSS had the following composition (in mM): NaCl 140, KCl 5, CaCl_2_ 2, MgCl_2_ 1, HEPES 20, and Glucose 10. For the preparation of high potassium solution (60 mM), NaCl was equimolarly replaced by KCl. The imaging set-up was built on a base of a fluorescence microscope Axio Observer A1 equipped with a Fluar 20×/0.75 objective (both Zeiss, Germany). Excitation at 340 nm and 380 nm (exposure time 100 ms) was achieved using a polychrome V monochromator (Till Photonics, Germany) and the emitted light with a cut off filter >500 nm was collected by a CMOS camera (ORCA-flash 4.0, Hamamatsu Photonics, Japan). The acquisition frequency was 1 Hz. The monochromator and the CMOS camera were controlled by the ZEN 2.0 Pro (Zeiss, Germany) acquisition software. Regions of interest (ROIs) were placed at cell somata and the fluorescence signals were background corrected. The time course of [Ca^2+^]_i_ changes was presented as F340/F380 ratio. All experiments were performed at room temperature.

Glutamate and Bafilomycin A1 were purchased from Sigma-Aldrich, Ned-19 was purchased from Santa Cruz Biotechnology. BZ194 was synthesized as described (Dammermann et al., 2009). After 5 min of baseline recording glutamate or bicuculline were applied and changes in [Ca^2+^]_i_ were monitored for 15 (glutamate) or 25 min (bicuculline) treatment. Thereafter, high-potassium solution (60 mM) was applied to identify excitable cells.

The change of basal [Ca^2+^]_i_ (Figures 5J,N,R) was calculated from the F 340/380 ratios at the time point 30 min subtracted by the F340/380 values at the time point 5 min.

### Gene Expression Analysis

Gene expression analysis was performed using the data of high throughput mRNA sequencing. Raw gene counts were taken from the gene expression omnibus (GEO) database under the accession numbers GSE104802 (Mao et al., 2018) and GSE142064 (unpublished^1^). For the unstimulated control samples of each dataset transcripts per kilobase million (TPM) were calculated and normalized to the mean mRNA expression value of the depicted genes from each dataset. Data processing and visualization were performed within the R environment (Version 1.2.5001).

### Statistical Analysis

Analysis of [Ca^2+^]_i_ peak amplitudes and area under the curve (AUC) was performed using Origin PRO software (Origin Lab 2015). Oscillation peaks observed under bicuculline treatment were classified as “low” or “high” if their absolute ratio amplitude was <0.15 or ≥ 0.15, respectively. The Fura-2 measurements from each independent cell preparation (made out of at least 4 embryos) were performed at least three times. 50–100 hippocampal neurons were measured simultaneously during each independent measurement. For calculation of a mean preparation trace, all the cells recorded with a particular protocol from a particular independent cell preparation were pooled. For the determination of significant differences of mean values obtained from two groups, a two-sample Student’s *t*-test was used (*p* < 0.05 for significance). *N* indicates the number of independent cell preparations and n indicates the number of measured cells unless otherwise stated.

## Results

### Current Concepts About Glutamate-Evoked and NAADP-Mediated Calcium Homeostasis in Neurons and Potential NAADP Target Channels

**FIGURE 1:**
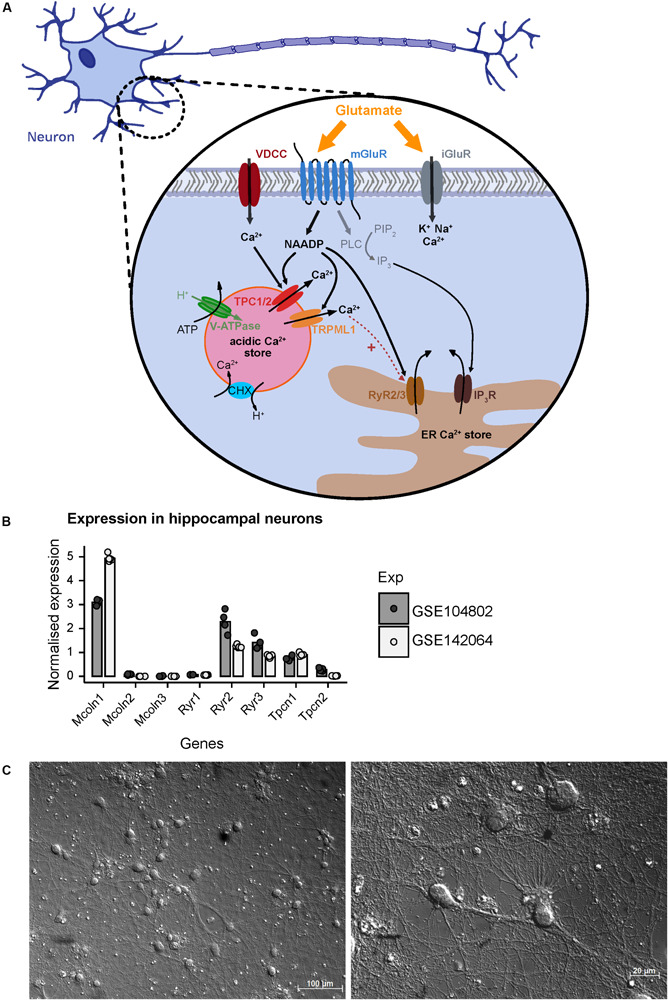
A model based on current literature summarizing regulators of NAADP-mediated Ca^2+^ homeostasis following extracellular glutamate action in hippocampal neurons of mice. **(A)** Glutamate activates ionotropic NMDA, AMPA, and KA receptors (iGluR) resulting in Ca^2+^ influx across the plasma membrane. The activation of metabotropic glutamate receptors (mGluR) induces the synthesis of inositol trisphosphate (IP_3_) which mediates Ca^2+^ release from the endoplasmic reticulum (ER). In the CNS and particularly in hippocampal neurons glutamate induces the synthesis of nicotinic acid adenine dinucleotide phosphate (NAADP) and subsequent Ca^2+^ release (Bak et al., 1999; McGuinness et al., 2007; Pandey et al., 2009). NAADP can be produced following activation of mGuR1 receptors and is known to mediate Ca^2+^ release into the cytosol via activation of two-pore channels 1/2 (TPC1/2) residing in the membrane of endo-lysosomal Ca^2+^ stores in many cell types including neurons (Bayraktar et al., 1990; Patel, 2015; Grimm et al., 2017; Foster et al., 2018) resulting in Ca^2+^ flux into the cytoplasm. Recently, it was also shown that Ca^2+^ entry via voltage gated Ca^2+^ channels triggers Ca^2+^ release from the lysosome via an NAADP-sensitive channel and, subsequently, fusion of the lysosome with the plasma membrane (Padamsey et al., 2017). In some cells the NAADP-evoked Ca^2+^ release depends on ryanodine receptors (RyR) such as the RyR1 in T cells (Diercks et al., 2018), and also TRPML1 channels were proposed as NAADP target channels (Zhang et al., 2011; Li et al., 2013). **(B)** Normalized mRNA expression of depicted genes in unstimulated primary hippocampal neuronal cultures. Transcripts per kilobase million (TPM) were calculated from GSE104802 (Mao et al., 2018; *n* = 4) and GSE142064 (*n* = 5) and were normalized to the mean expression from each dataset. **(C)** Representative images of primary hippocampal neurons of a C57BL/6N mice after 14 days *in vitro* as used in this study; photomicrography was performed using a transmitted light microscope with differential interference contrast (upper figure: objective EC Plan-Neofluar 20×/0.50 M27; lower figure: Plan Apochromat 63×/1.4 Oil DIC M27). Scale bars 100 μM, 20 μM.

In addition to ionotropic NMDA, AMPA, and KA receptors, glutamate activates mGluRs to elicit downstream signaling events in the cell (Reiner and Levitz, 2018). It was shown that stimulation of hippocampal neurons with glutamate triggers NAADP synthesis probably via activation of mGluR1 receptors and mediates calcium release from lysosomal stores via two-pore-channels (Bak et al., 1999; McGuinness et al., 2007; Pandey et al., 2009; Foster et al., 2018). The prerequisite for this process is the loading of acidic stores with calcium ions which depends on the activity of the V-ATPase (Figure 1) and acidification that then allows Ca^2+^ uptake via H^+^/Ca^2+^ exchange. Recently, experimental evidence was reported that calcium entry into neurons mediated by voltage gated calcium channels can trigger calcium release from acidic stores via NAADP sensitive channels. In addition to Two Pore Channel type 1 (TPC1) and TPC2 channels (Rahman et al., 2014; Grimm et al., 2017), TRPML1 (Zhang et al., 2011), and ryanodine receptors such as RYR1 have been reported as a NAADP target channels in other cell types (Guse, 2012; Diercks et al., 2018). The current concepts of NAADP-mediated calcium release and potential NAADP target channels that also may operate in hippocampal neurons are summarized in Figure 1A based on two independent RNAseq expression analyses in primary cultured neurons from mouse hippocampus GSE104802 (Mao et al., 2018) and GSE142064 (unpublished). Our analysis of these studies revealed that transcripts of Mcoln1 (encoding TRPML1 channels) are abundantly expressed in murine hippocampal neuron cultures whereas Mcoln2 and Mcoln3 transcripts are virtually not detectable. Within the TPC channel family, Tpcn1 transcripts (encoding TPC1) were detected (Figure 1B) and Tpcn2 transcripts (encoding TPC2) were also present, however, to a lower extent. Amongst the ryanodine receptors, transcripts encoding Ryr2 and Ryr3 were readily detected, whereas expression of Ryr1 seems to be negligible in hippocampal neurons in both studies.

In the current study we aim to analyze the contribution of NAADP-mediated calcium release from acidic stores in primary hippocampal neurons. Representative images of cultured hippocampal neurons 14 days after isolation, which were analyzed in our study, are shown in Figure 1C and demonstrate a dense and complex network of synaptic connections between the neurons. Cell bodies are 13–20 μm in size, from which numerous axonal structures originate similarly as has been described in previous publications (Mao and Wang, 2001). We have studied a possible involvement of NAADP-mediated Ca^2+^-release in [Ca^2+^]_i_ elevation events registered after extracellular application of glutamate or during endogenous glutamate release events triggered by inhibition of GABA_A_ receptors. To this end, we tested different concentration of glutamate (1, 3, and 10 μM) and measured the time course of intracellular calcium rise using Fura-2-based microfluorimetry. The rise of [Ca^2+^]_i_ triggered by 1 μM glutamate was transient and completely returned to the baseline ∼5 min after the beginning of stimulation (Supplementary Figure 1A), whereas the responses elicited by application of 3 μM glutamate had a higher amplitude and a much longer decay phase with a half-decay time from about ∼8 min (Supplementary Figure 1B). Following application of 10 μM glutamate the calcium plateau did not revert within a time frame of 15 min (not shown). Thus, protocols using the application of 3 μM glutamate were used in the following to test its modulation by interference with NAADP antagonists.

### Glutamate Evoked [Ca^2+^]_i_ Rise in Hippocampal Neurons Is Reduced by NAADP Antagonists

**FIGURE 2:**
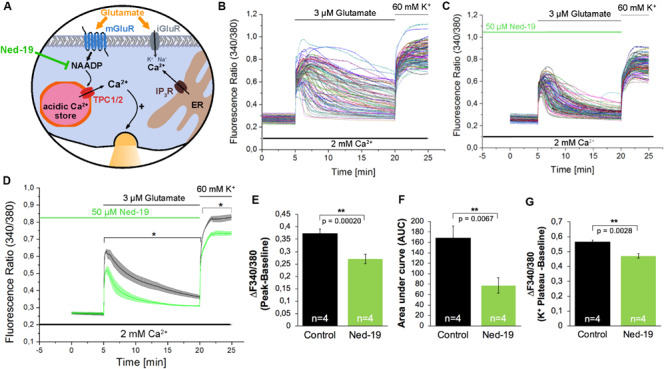
The NAADP antagonist Ned-19 reduces the glutamate-induced Ca^2+^ increase in hippocampal neurons. **(A)** Ned-19 acts as a NAADP antagonist (Naylor et al., 2009). **(B,C)** Representative traces of the glutamate-induced changes in the F340/380 fluorescence intensity values in hippocampal neurons without **(B)** and with **(C)** pretreatment with Ned-19 (50 μM). **(D)** Average traces are given as the arithmetic mean ± SEM of the F340/380 fluorescence intensity values without (black, *n* = 1057 cells) and with (green, *n* = 659 cells) Ned-19 (50 μM) pre-incubation. Data from four independent C57Bl/6N preparations (*N* = 4) are shown. During all experiments, the cells were remained in physiological solution containing 2 mM Ca^2+^. For measurements with Ned19, the cells were pre-incubated for 5 min. After 5 min of baseline recording, the cells were stimulated with glutamate (3 μM) for 15 min. For the last 5 min, the cells were stimulated with high potassium solution (60 mM K^+^). The amplitude of the glutamate-induced calcium increase **(E)** as well as the AUC **(F)** and the amplitude of the high-potassium induced calcium transients **(G)** were analyzed. **: *p* < 0,01; *: *p* < 0,05.

After Fura-2 loading, hippocampal neurons were pre-incubated with Ned-19 (50 μM) for 5 min before starting the recording. 5 min after beginning of the recording, the cells were stimulated with glutamate (3 μM) for 15 min. Finally, to exclude possible recording of [Ca^2+^]_i_ in glial cells, we stimulated the cells with high-potassium solution (60 mM) as a positive control for excitable cells. Representative recordings are shown in Figure 2B and demonstrate an instantaneous [Ca^2+^]_i_ rise followed by a continuous and more variable decay phase. In average, the fluorescence ratio declined during 15 min after glutamate application to ∼30% of initial amplitude (Figure 2D, black traces).

The treatment with Ned-19 resulted in a significant reduction of the amplitude of glutamate-evoked [Ca^2+^]_i_ rise (Figures 2C–E). The F340/F380 ratio was reduced by ∼27%. We also observed a ∼54% reduction of the AUC of the fluorescence ratio for the time period of 15 min after glutamate application (Figure 2F). After application of high potassium solution all cells in both conditions reacted with fast [Ca^2+^]_i_ rise reaching a plateau phase about 2 min after the stimulation. However, the F340/F380 fluorescence ratio amplitude of this high potassium-induced responses was reduced by ∼16% in cells pretreated with the NAADP antagonist Ned-19 (Figure 2G).

**FIGURE 3:**
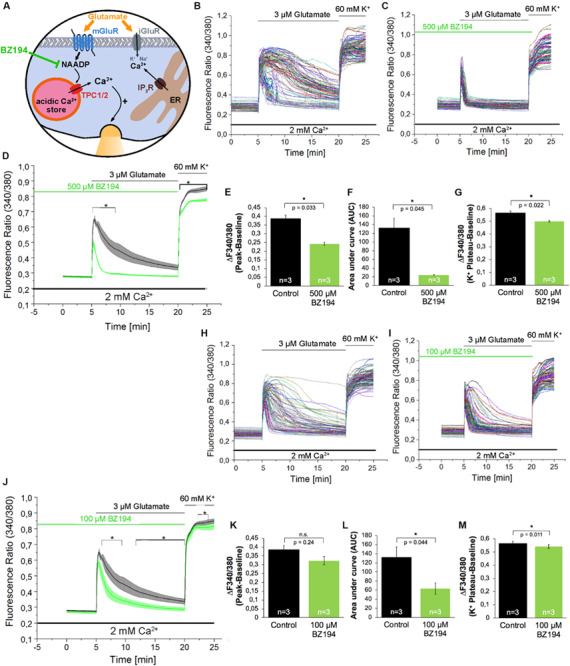
The effect of the NAADP antagonist BZ194 on the time course of the glutamate-induced Ca^2+^ increase in hippocampal neurons. **(A)** BZ194 acts as a NAADP antagonist (Dammermann et al., 2009). **(B,C,H,I)** Representative traces of the glutamate-induced changes in the F340/380 fluorescence intensity values of hippocampal neurons without **(B,H)** and with 500 μM **(C)** or 100 μM **(I)** pre-incubation with BZ194. **(D,J)** Average traces are given as the arithmetic mean ± SEM of the averaged F340/380 fluorescence intensity values without (black, *n* = 719 cells in **D** and **J**) and with (green) 500 μM (**D**, *n* = 650 cells) or 100 μM (**J**, *n* = 611 cells) BZ194 pre-incubation. Data from independent C57Bl/6N preparations (*n* = 4) are shown. During all experiments, the cells were remained in physiological solution containing 2 mM Ca^2+^. For measurements with BZ194, the cells were pre-incubated for 5 min. After 5 min recording, the cells were stimulated with glutamate (3 μM) for 15 min. For the last 5 min, the cells were stimulated with high potassium solution (60 mM K^+^). Finally, the amplitude of the glutamate-induced calcium increase **(E,K)** as well as the AUC **(F,L)** and the amplitude of the high-potassium induced calcium transients **(G,M)** were analyzed. **: *p* < 0,01; *: *p* < 0,05.

In addition to Ned-19, we used BZ194 as an independent NAADP antagonist. BZ194 was shown to effectively reduce NAADP-dependent calcium transients at a concentration of 500 μM (Nebel et al., 2013) and (Figure 3A). As it is evident from the representative traces (Figures 3B,C) and the average traces (Figure 3D), it is obvious that BZ194 pretreatment at the concentration at 500 μM does not prevent immediate [Ca^2+^]_i_ rise although the peak ratio levels were reduced significantly by ∼38% (Figure 3E), and the AUC of the [Ca^2+^]_i_ transients as a measure for the total calcium elevation over the period of stimulation is largely reduced by ∼82% (Figure 3F). Similarly, like in case of Ned-19 (50 μM) pretreatment the amplitude of high potassium-evoked [Ca^2+^]_i_ transients was also reduced. The observed reduction was ∼12% (Figure 3G). As the NAADP antagonist BZ194 was shown to affect calcium release in a dose-dependent manner in *T* lymphocytes and cardiomyocytes (Dammermann et al., 2009; Nebel et al., 2013) we performed similar experiments with BZ194 pretreatment in hippocampal neurons using a lower concentration (100 μM, Figures 3H–M). Under these conditions the inhibition of glutamate evoked [Ca^2+^]_i_ rise was less pronounced as compared to BZ194 applied at the concentration of 500 μM. The AUC of the [Ca^2+^]_i_ transient was reduced by ∼52% (Figure 3L) and the amplitude of glutamate evoked [Ca^2+^]_i_ rise was partially reduced in average, but was not significantly different from the non-treated cells (Figure 3K). High potassium-evoked calcium rise was still significantly reduced at some time points but the reduction was only ∼5% in average (Figure 3M).

**FIGURE 4:**
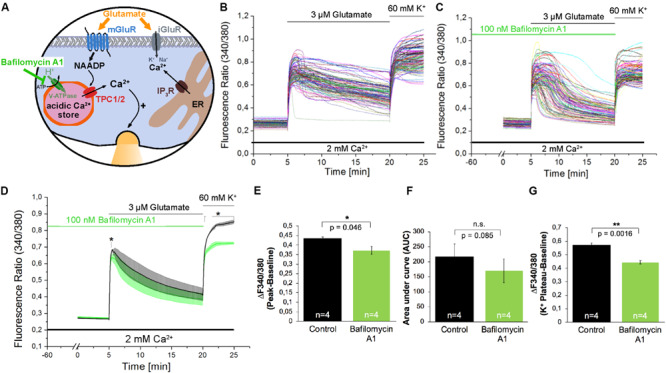
The V-ATPase blocker Bafilomycin A1 does not affect glutamate-induced Ca^2+^ increase in hippocampal neurons. **(A)** Model depicting the action of Bafilomycin A1 as inhibitor of H^+^ transport into acidic organelles. **(B,C)** Representative traces of the glutamate-induced changes in the F340/380 fluorescence intensity values in hippocampal neurons without **(B)** and with **(C)** pretreatment with Bafilomycin A1 (100 nM). **(D)** Average traces are given as the arithmetic mean ± SEM of the averaged F340/380 fluorescence intensity values without (black, *n* = 745 cells) and with (green, *n* = 849 cells) Bafilomycin A1 (100 nM) pre-incubation. Data from independent C57Bl/6N preparations (*N* = 4) are shown. During all experiments, the cells were remained in physiological solution containing 2 mM Ca^2+^. For measurements with Bafilomycin A1, the cells were pre-incubated for 60 min. After 5 min of baseline recording, the cells were stimulated with glutamate (3 μM) for 15 min. For the last 5 min, the cells were stimulated with high potassium solution (60 mM K^+^). The amplitude of the glutamate-induced calcium increase **(E)** as well as the AUC **(F)** and the amplitude of the high-potassium induced calcium transients **(G)** were analyzed. **: *p* < 0,01; *: *p* < 0,05.

The acidification of endo-lysosomes depends on the activity of the Vacuolar-type H^+^-ATPase (V-ATPase; Figure 4A), and by its inhibition using Bafilomycin A1 (Bowman et al., 1988) the calcium accumulation in the acidic stores can be prevented indirectly by inhibition of lysosomal acidification in many cell types (Yoshimori et al., 1991; Shen et al., 2012). Pre-incubation with Bafilomycin A1 (100 nM) for 60 min only slightly reduced the amplitude of glutamate evoked [Ca^2+^]_i_ transients by ∼16% (Figures 4B–E) and the AUC of these responses was not significantly reduced under the conditions tested (Figure 4F). Nevertheless the fluorescence ratio amplitude of the high potassium solution-evoked responses was significantly reduced by ∼23% (Figure 4G).

### Calcium Transients Triggered by Bicuculline-Evoked Glutamate Release Events Are Modulated by NAADP Antagonists

**FIGURE 5:**
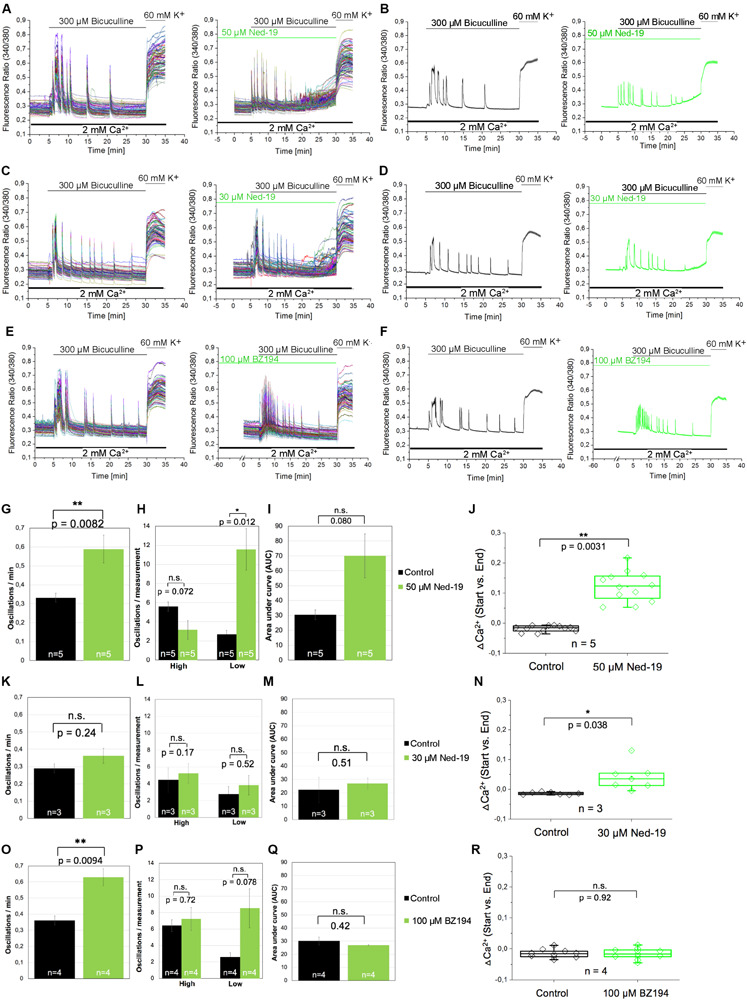
The effect of the NAADP antagonists Ned19 and BZ194 on Ca^2+^ transients triggered by glutamate release events following inhibition of GABA_A_ receptors in hippocampal neurons. **(A,C,E)** Representative traces of the bicuculline-induced changes in the F340/380 fluorescence intensity values in hippocampal neurons without (left) and with (right) preincubation with Ned-19 (50 μM; **A** or 30 μM; **C**) or BZ194 (100 μM; **E**). **(B,D,F)** Average traces are given as the arithmetic mean ± SEM without (black) and with (green) the corresponding NAADP antagonists (50 μM Ned-19, *n* = 89 cells, **B**; 30 μM Ned-19, *n* = 86 cells, **D**; and 100 μM BZ194, *n* = 79 cells, **F**). During all experiments, the cells were remained in physiological solution containing 2 mM Ca^2+^. For measurements with Ned19, the cells were pre-incubated for 5 min; for measurements with BZ194, the cells were pre-incubated for 60 min. After 5 min recording, the cells were stimulated with bicuculline (300 μM) for 25 min. For the last 5 min, the cells were stimulated with high potassium solution (60 mM K^+^). For each antagonist, several independent preparations (50 μM Ned19: *N* = 5: upper line; 30 μM Ned19: *N* = 3: middle line; and 100 μM BZ194: *N* = 4: lower line) were pooled. The oscillation frequency **(G,K,O)**, the number of high and low peaks **(H,L,P)** as well as the AUC **(I,M,Q)** were determined for each preparation without (black) and with (green) pre-incubation of the respective NAADP antagonist. **(J,N,R)** The change of the intracellular Ca^2+^ levels before and 25 min after bicuculline application (ΔCa^2+^) was determined for each measurement without (black) und with (green) pre-incubation of the respective NAADP antagonist. For each p-value: *n* = 5 (50 μM Ned19); *n* = 3 (30 μM Ned19), and *n* = 4 (100 μM BZ194). **: *p* < 0,01; *: *p* < 0,05.

In the next set of experiments, we aimed to investigate whether NAADP not only affects the calcium transients evoked by extracellular application of glutamate, but also by glutamate release events that occur endogenously in cells of our neuronal cultures. To facilitate such glutamate release events the cells were treated with bicuculline, a GABA_A_ receptor antagonist, to prevent the inhibitory influence of these chloride channels on exocytosis of neurotransmitters (Hardingham et al., 2002). To this end, bicuculline (300 μM) was applied 5 min after recording of the resting [Ca^2+^]_i_. Representative traces of individual cells as well as the average traces are shown in Figures 5A–F. The bicuculline (300 μM) evoked changes in [Ca^2+^]_i_ appear as repetitive Ca^2+^ oscillations, were evoked within the first minute after bicuculline application and appeared synchronized between all tested cells of the culture dish. To assess whether NAADP antagonists affect the properties of these synchronized calcium transients, we quantified the oscillation frequency (Figures 5G,K,O), the AUC (Figures 5I,M,Q) as well as the amplitude of the corresponding calcium transients. To this end we compared calcium transients with high and low amplitudes (see method section for details) in bicuculline-evoked calcium transients with and without NAADP antagonist pretreatment (Figures 5H,L,P). In these experiments, cells were pretreated with Ned-19 in a concentration of 50 μM (Figures 5A,B), or 30 μM (Figures 5C,D) for 5 min. BZ194 (100 μM, Figures 5E,F) was pre-incubated for 60 min. The quantitative analysis of the time course of bicuculline-evoked calcium transients showed that pre-incubation with Ned-19 (50 μM) and BZ194 (100 μM), respectively, significantly altered the properties of bicuculline-evoked calcium transients, whereas Ned-19 at the concentration of 30 μM had no significant effect. In fact, the oscillation frequency was significantly increased by both NAADP antagonists (Figures 5G,O). Ned-19 (50 μM) pretreatment led to a significant increase in the number of calcium transients with a lower amplitude at the cost of transients with a higher amplitude although the reduction of the proportion of higher amplitudes itself was not statistically significant (Figure 5H). In average, there was a tendency toward an increase of the AUC upon Ned-19 (50 μM) pretreatment (Figure 5I). We noticed that the [Ca^2+^]_i_ continuously increased in most experiments particularly at the end of the observation time between 25 and 30 min after bicuculline application when cells were pretreated with Ned-19 (50 μM; Figures 5A,B, right panels). Therefore, we quantified the average change in [Ca^2+^]_i_ at the end of the bicuculline treatment compared to the calcium levels before bicuculline applications in cells without (black) and with Ned-19 (50 μM, green) pretreatment (Figure 5J). We found that Ned-19 treatment caused a significant calcium accumulation over this observation time period under bicuculline action and this increase in resting [Ca^2+^]_i_ was also observed to some degree when Ned-19 pretreatment was performed at the concentration of 30 μM, however, a similar effect was not observed with BZ194 (100 μM) pretreatment.

## Discussion

Over the last two decades, the concept of generation of NAADP in neurons following stimulation with glutamate has been first developed in brain microsomes (Bak et al., 1999) and then in lysosome-related organelles in hippocampal neurons (McGuinness et al., 2007). The generation of NAADP upon extracellular glutamate application has been directly measured (Pandey et al., 2009), however, the contribution of lysosomal stores due to glutamate evoked Ca^2+^ release relied on the depletion of lysosomal calcium stores by the dipeptide glycyl-l-phenylalanine 2-naphthylamide (GPN) that prevents organellar acidification. Yet, a recent study has demonstrated that GPN-evoked increase in [Ca^2+^]_i_ is not due to selective targeting of GPN on lysosomes, but increases cytosolic pH and subsequently Ca^2+^ release from the ER (Atakpa et al., 2019). However, our results using NAADP antagonists show that NAADP signaling contributes to glutamate-evoked [Ca^2+^]_i_ rise after extracellular glutamate application in hippocampal neurons. We also find that the nature of calcium transients, which occur in synchronous manner in all neurons throughout culture of hippocampal neurons after endogenous glutamate release, also depends on NAADP signaling as revealed by the use of the NAADP antagonists.

To analyze the contribution of NAADP-evoked signaling events during glutamate evoked [Ca^2+^]_i_ rise, in the present study, we used two independent NAADP antagonists, Ned-19 (Naylor et al., 2009), and BZ194 (Dammermann et al., 2009). Pre-incubation with Ned-19 (50 μM) reduced peak amplitudes of glutamate evoked [Ca^2+^]_i_ rise by ∼27%, after pretreatment with BZ194 (500 μM) peak amplitudes of glutamate-evoked calcium transients decreased by ∼38%. In previous research, Ned-19 was used at this concentration in other cell types to antagonize NAADP action (Esposito et al., 2011), and BZ194 at this concentration was found to significantly reduce the Isoproterenol-evoked [Ca^2+^]_i_ rise from acidic stores in cardiomyocytes (Nebel et al., 2013). Pre-incubation with Bafilomycin A1, a V-ATPase blocker, which erases calcium accumulation in acidic stores indirectly by preventing their acidification (Bowman et al., 1988; Yoshimori et al., 1991), resulted only in a ∼16% reduction of the amplitude of glutamate-evoked Ca^2+^ release. In a previous study, the Bafilomycin A1 effect on glutamate evoked [Ca^2+^]_i_ rise amplitude was more pronounced reaching ∼50% reduction of the Ca^2+^ transient amplitude, however, in the aforementioned study, Bafilomycin A1 was used in a much higher concentration (1 μM vs. 100 nM in this study) and glutamate was applied at a concentration of 10 μM to stimulate the cells (Pandey et al., 2009). Another reason behind this discrepancy might also be that this study analyzed calcium transients in the absence of extracellular calcium levels, which was not pursued in our study, as the removal of extracellular calcium ions repeatedly triggered spontaneous calcium transients in mouse hippocampal neurons (data not shown). In addition, the fact that glutamate evokes Ca^2+^ entry also via ionotropic glutamate receptors in the presence of extracellular Ca^2+^, as well as via voltage-gated calcium channels (Bading et al., 1993; Hardingham et al., 2001), can also contribute to the differences in the effectivity of Bafilomycin A1 between the two studies. Nevertheless, our study, using two independent NAADP antagonists and Bafilomycin A1, provide evidence about the contribution of NAADP-mediated calcium release and acidic stores to the amplitude of glutamate-evoked global calcium rise in hippocampal neurons of the mouse. Additional evidence comes from the analysis of the AUC of the glutamate-evoked [Ca^2+^]_i_ rise as Ned-19 (50 μM) pretreatment reduced AUC by ∼54%, and pretreatment with BZ194 reduced AUC by ∼82% and ∼52% when used at concentrations of 500 μM and 100 μM, respectively. Bafilomycin A1 did not affect this parameter, which can be attributed to the differences in the kinetics, and/or in the ability to evoke alkalization of acidic calcium stores by Bafilomycin A1 in the mouse and rat’s hippocampal neurons. There could be some differences in effectivity of Bafilomycin A1 depending on cell type and time window (Klionsky et al., 2008).

Stimulation of the neuronal cultures with a high potassium solution led to an instantaneous increase that in contrast to glutamate stimulation was characterized by a continuous plateau. This response is similar to previously reported findings (Verkhratsky and Petersen, 2010). Interestingly, the high potassium-evoked [Ca^2+^]_i_ rise, which is supposed to be mediated by voltage gated calcium channels, was significantly reduced by both NAADP antagonists, Ned-19 and BZ194, as well as by pretreatment with the V-ATPase inhibitor Bafilomycin A1. A possible explanation for this result is that calcium entry via voltage-gated calcium channels can lead to a subsequent activation of Ca^2+^ release from NAADP-sensitive lysosomal calcium stores (Padamsey et al., 2017). An alternative explanation can be that endo-lysosomal calcium release supports the trafficking of *N*-type calcium channels into the plasma membrane (Hui et al., 2015). In agreement with the results reported by Padamsey et al. (2017) and Hui et al. (2015), the functional inactivation of endo-lysosomal calcium stores, which we performed in the present study using NAADP antagonists and Bafilomycin A1, would disrupt mutually facilitating interaction between lysosomal Ca^2+^ release and voltage-dependent calcium entry resulting in the reduced depolarization-induced calcium entry (see Figures 3D,J, 4D). However, it remains to be determined in further research whether the Ca^2+^ release from the acidic stores precedes the activation of voltage-gated channels or vice versa.

In order to corroborate the relevance of NAADP signaling found after extracellular application of glutamate in micromolar concentrations we aimed to study conditions that are more similar to those occurring *in vivo* where glutamate is released locally by exocytotic release, e.g., in the synaptic cleft or even extrasynaptically. To this end, we performed experiments in which we applied the GABA_A_ receptor antagonist bicuculline. This experimental strategy has been commonly used to remove the inhibitory influence of the GABA_A_-mediated chloride currents on the exocytosis in glutamatergic neurons. Bicuculline-evoked calcium signals were characterized by [Ca^2+^]_i_ oscillations synchronized through all neuronal cells in the cell culture dish. Both Ned-19 (50 μM) and BZ194 (100 μM) pretreatments resulted in a significant increase in the frequency of bicuculline-evoked [Ca^2+^]_i_ oscillations, accompanied by a significant increase in the occurrence of calcium transients with a lower amplitude. While further research is needed to identify physiological and pathophysiological consequences of such changes in the nature of these glutamate-evoked [Ca^2+^]_i_ oscillations, it is already known that a precise regulation of Ca^2+^ release from acidic stores via two-pore channels is essential for regulation of synaptic plasticity, since deletion of TPC channels leads to a reversal from long-term potentiation to long-term depression in mice (Foster et al., 2018).

### Potential NAADP Target Channels in Hippocampal Neurons

To get an idea which channels might be present in our cell system to mediate glutamate-evoked calcium release we analyzed the expression of candidate NAADP target channels in two independent RNA seq experiments that were performed in murine hippocampal neurons, i.e., GSE104802 (Mao et al., 2018) and GSE142064 (unpublished). The analysis suggests that both TPC channel subtypes, TPC1 and TPC2, were expressed in murine hippocampal neurons and might operate as NAADP target channels upon glutamate stimulation similarly like TRPML1. The causal contribution of any of these channels could be revealed by studying glutamate evoked Ca^2+^ transients in hippocampal neurons that are deficient for the corresponding channels or by patch clamp recordings performed in the endo-lysosomes of these cells as was done before in other cell types (Chen et al., 2017). Alternatively, agonists and antagonists that specifically target individual channel subtypes, as recently reported for members of these channel families (Plesch et al., 2018; Gerndt et al., 2020), might be useful tools to pinpoint the contribution of distinct channel entities in this process. TRPML1 protein expression in cultured mouse embryonic hippocampal neurons was previously demonstrated using Western blotting analysis (Zhang et al., 2017). RYR1, which operates as an NAADP target channel early during *T* cell activation (Wolf et al., 2015; Diercks et al., 2018), seems to be not expressed in cultured murine hippocampal neurons. In contrast to RYR2 and RYR3, we did not find RYR1 expression in mouse embryonic hippocampal neurons. Similar results were reported in the study analyzing developmental changes in the expression of the three ryanodine receptor mRNAs in the mouse brain (Mori et al., 2000). These authors report that in the hippocampal CA1 region RYR1 mRNA appeared only at P1 and reached its peak level at P7. Also in the adult mouse hippocampal CA region, RYR1 had the lowest expression level compared to RYR2 and RYR3. A contribution of RYR2 and/or RYR3 for intracellular Ca^2+^ rise downstream of NAADP generation has not been reported so far, although these receptors appear to be regulated by cyclic ADP-ribose (Fritz et al., 2005; Dabertrand et al., 2007). However, RYR2 and RYR3 should be studied in the future with similar approaches as discussed for TPC and TRPML1 channels above.

Taken together, the results of this study provide evidence that NAADP-sensitive calcium stores contribute to the alteration in cellular calcium homeostasis in neurons evoked by glutamate. Whether calcium release from these acidic stores aggravates excitotoxicity in disease models with inflammatory neuronal degeneration such as Multiple Sclerosis needs to be elaborated in further research by the inhibition of endo-lysosomal channels with specific antagonists or their genetic deletion.

## Data Availability Statement

The raw data supporting the conclusions of this article will be made available by the authors, without undue reservation, to any qualified researcher.

## Ethics Statement

The animal study was reviewed and approved by Regierungspräsidium Karlsruhe and the University Heidelberg.

## Author Contributions

MF and VT: Conceptualization. JH and MW: Data curation. JH, VT, MW, VK, and MF: Formal analysis. MAF and MF: Funding acquisition. JH, VT, and MF: Investigation. JH, MB, DS, MW, AS, SR, and VT: Methodology. VT, MAF, and MF: Project administration. CM and AG: Resources. VT, MAF, and MF: Supervision. JH, MW, and MF: Visualization. JH, VT, and MF: Writing – original draft. Writing – review and editing, all authors.

## Conflict of Interest

The authors declare that the research was conducted in the absence of any commercial or financial relationships that could be construed as a potential conflict of interest.

## Footnotes

1 https://www.ncbi.nlm.nih.gov/geo/query/acc.cgi?acc=GSE142064
