# Supplementary figures and images for: Contribution of NAADP to Glutamate-Evoked Changes in Ca2+ Homeostasis in Mouse Hippocampal Neurons
Source: Front Cell Dev Biol. 2020 Jun 25;8:496. doi: 10.3389/fcell.2020.00496 (PMC7333232; doi:10.3389/fcell.2020.00496)

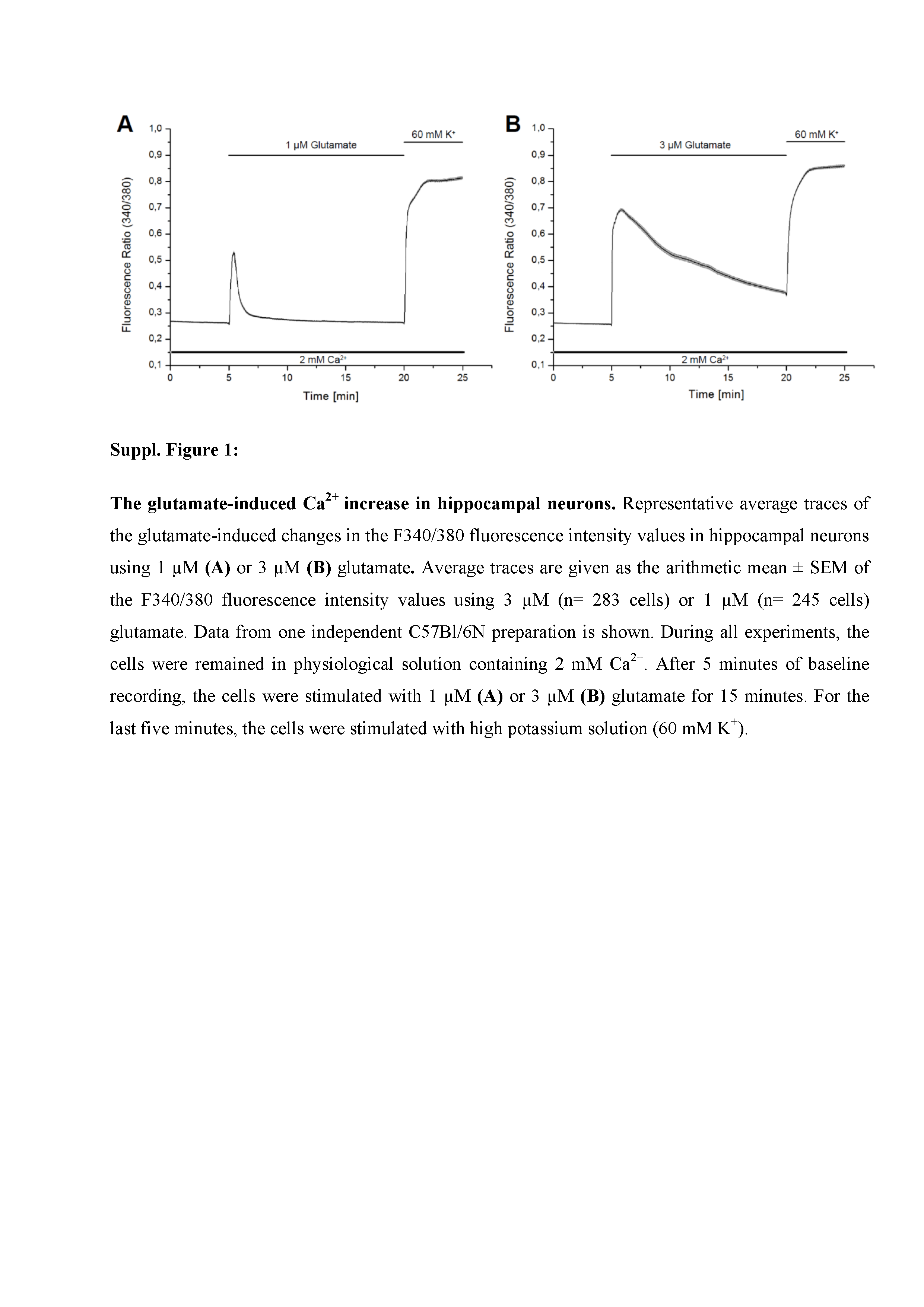

Supplement: Supplementary file 1 [file Image_1.TIFF]
